# Spindle Cell Carcinoma of the Larynx Arising From Aggressive Fibromatosis

**DOI:** 10.7759/cureus.74717

**Published:** 2024-11-29

**Authors:** Vedika Dhunnoo, Richard Stevens, Andrew Kinshuck

**Affiliations:** 1 Otolaryngology - Head and Neck Surgery, Liverpool University Foundation Trust, Liverpool, GBR; 2 Otolaryngology - Head and Neck Surgery, Aintree University Hospital, Liverpool, GBR

**Keywords:** carcinoma of larynx, laryngeal fibromatosis, masslike lesion in the larynx, otolaryngology, spindle cell neoplasm

## Abstract

Aggressive fibromatosis is a rare, benign proliferative disease with unknown aetiology and high recurrence rate. To date, there are only eight reported cases affecting the larynx. Four were managed with total laryngectomy, whilst spontaneous regression happened in one case. Spindle cell carcinoma is a rare but highly aggressive biphasic tumour which often arises in the head and neck.

Diagnosed with laryngeal fibromatosis more than 10 years ago, our patient had undergone six trans-oral laser debulking procedures. On this occasion, he presented with a two-week history of severe worsening dyspnoea and hoarseness of voice, requiring urgent debulking. Final pathology results revealed spindle cell carcinoma of the larynx.

In this report, we describe the case of laryngeal fibromatosis which progressed to spindle cell carcinoma. To our knowledge, no such case has been described previously. This case highlights an important complication of laryngeal fibromatosis and emphasises the importance of regular follow-ups.

## Introduction

Desmoid tumours, also known as aggressive fibromatosis, are defined as benign, fibroblastic neoplasms that arise from deep musculoaponeurotic structures [1]. They are rare, accounting for only around 0.03% of all tumours [1]. Desmoid tumours do not metastasise; however, they cause local infiltration and tend to recur [2]. Most cases arise in the abdominal region, whilst extra-abdominal fibromatosis frequently occurs in the head and neck region, with the larynx being an exceptionally rare site [3-4]. To date, only eight cases of laryngeal fibromatosis have been described in the literature [4-6]. Due to the rarity of the disease, studies describing the management of patients with aggressive fibromatosis are limited [7].

Spindle cell carcinoma is a rare type of malignancy that often originates in the head and neck region, namely the larynx and the hypopharynx [8]. It is a more aggressive and poorly differentiated version of squamous cell carcinoma [8]. Previously, cases of spindle cell carcinoma were often misdiagnosed, but today, advances in molecular and histological techniques have enabled better recognition of the disease [9]. The mainstay treatment for this disease is surgery; however, currently, no standard protocol is available [10].

Here, we report a case of an octogenarian gentleman with laryngeal fibromatosis which progressed into spindle cell carcinoma.

## Case presentation

An octogenarian retired ex-smoker had an urgent admission at a tertiary otolaryngology department due to a two-week history of worsening dyspnoea and hoarseness of voice. The patient was first diagnosed with laryngeal fibromatosis more than 10 years ago. He previously underwent trans-oral laser resection and had been symptom-free for eight consecutive years. Apart from pleural plaques, the patient did not have any other significant co-morbidities.

Nasendoscopic examination revealed significant recurrence of the fibromatosis lesion at the anterior commissure as well as posteriorly. He was listed for an urgent microlaryngobronchoscopy, laser resection and keel insertion.

**Figure 1:**
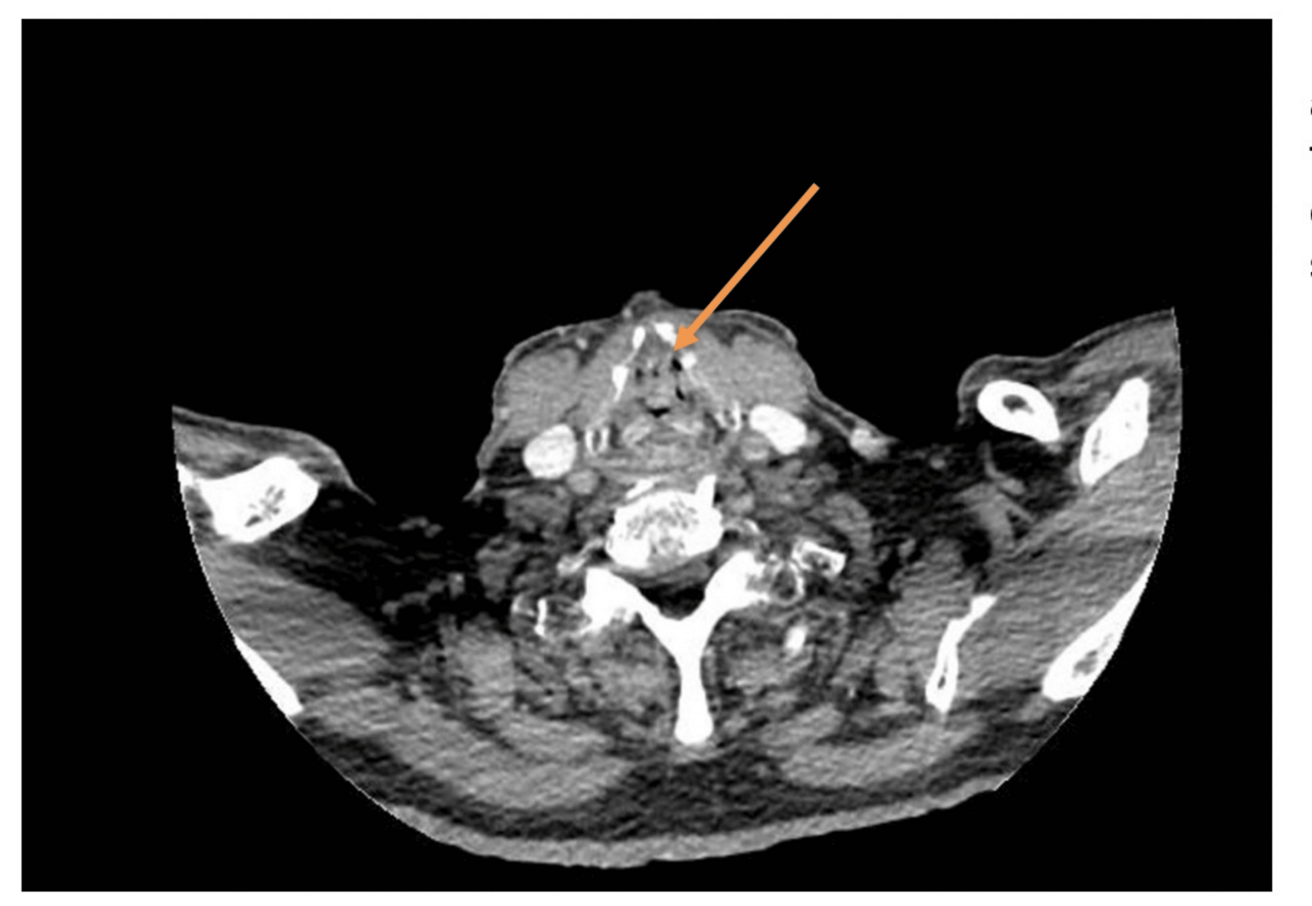
CT scan taken on admission showing soft tissue filling defect in the glottic and supraglottic airway. The arrow denotes the soft tissue filling defect in the glottic and supraglottic airway.

On admission, a CT scan (Figure 1) was performed, which showed a soft tissue filling defect in the glottic and supraglottic airway, with almost complete airway obstruction at this level. There was no evidence of extra laryngeal fibromatosis. A subsequent staging CT of the chest was clear.

A year prior to this admission, the patient underwent an MRI scan, which did not show any definitive focal lesion within the larynx.

On his first presentation, more than 10 years ago, biopsy samples were taken from a lesion on the posterior end of the right vocal cord. Histology revealed hyperplastic mucosa with hyperkeratosis and collagenous tissue and confirmed aggressive fibromatosis.

**Figure 2:**
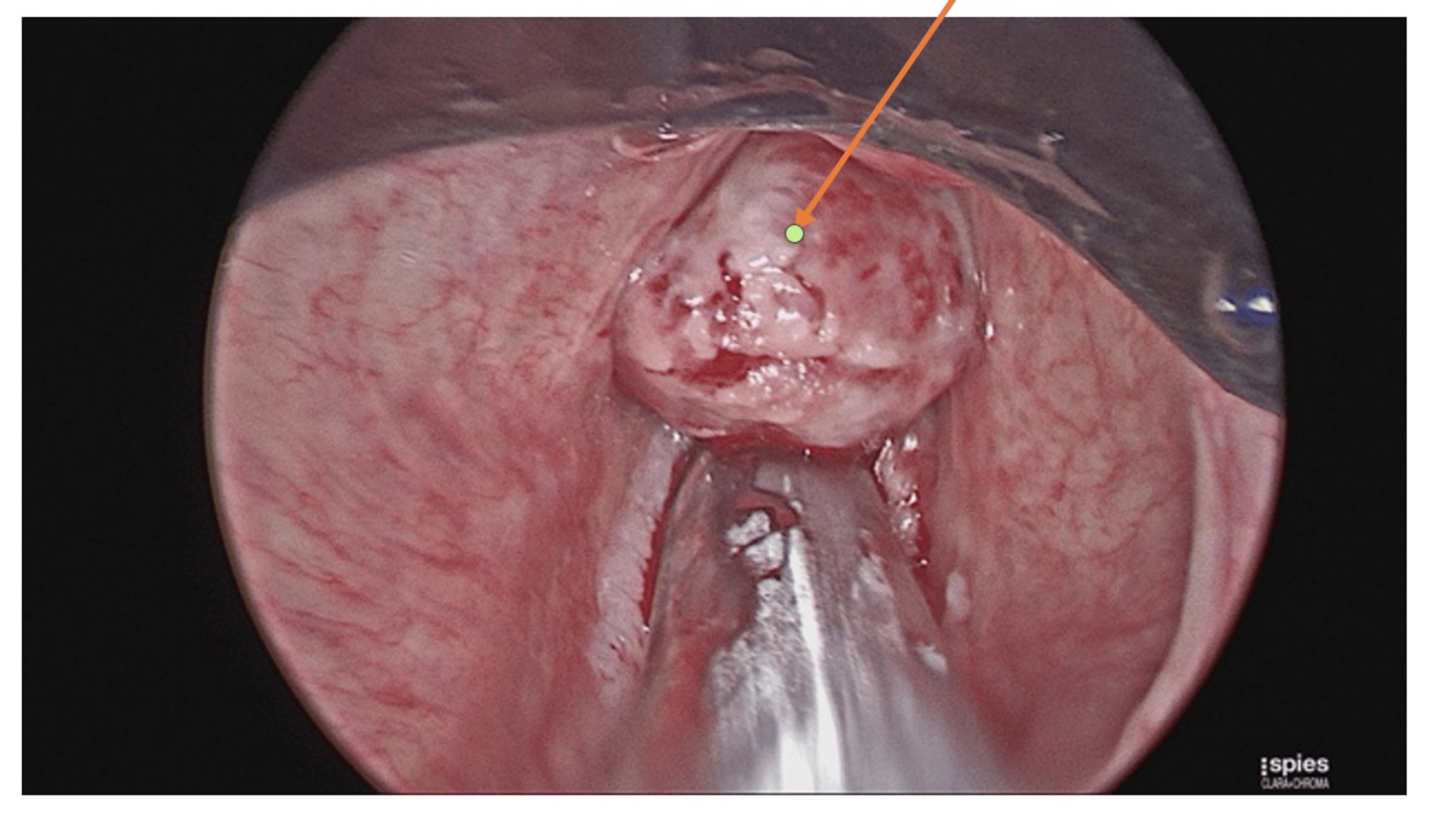
Laryngeal mass in the glottic and supraglottic airway visualised on microlaryngobronchoscopy. The arrow denotes the laryngeal mass.

**Figure 3:**
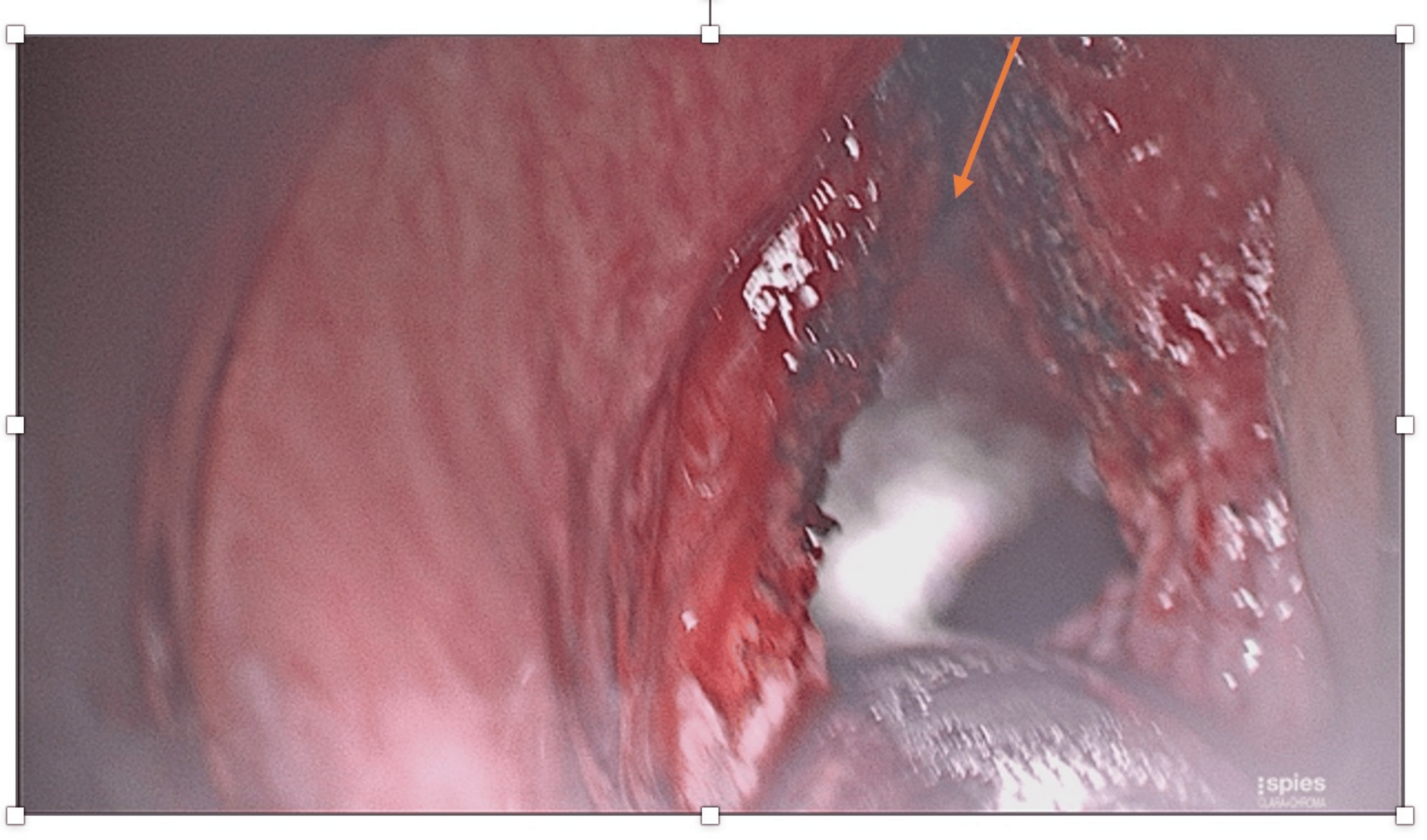
Post-laser debulking of the laryngeal mass. The arrow denotes the area where the laryngeal mass was present prior to the debunking process.

**Figure 4:**
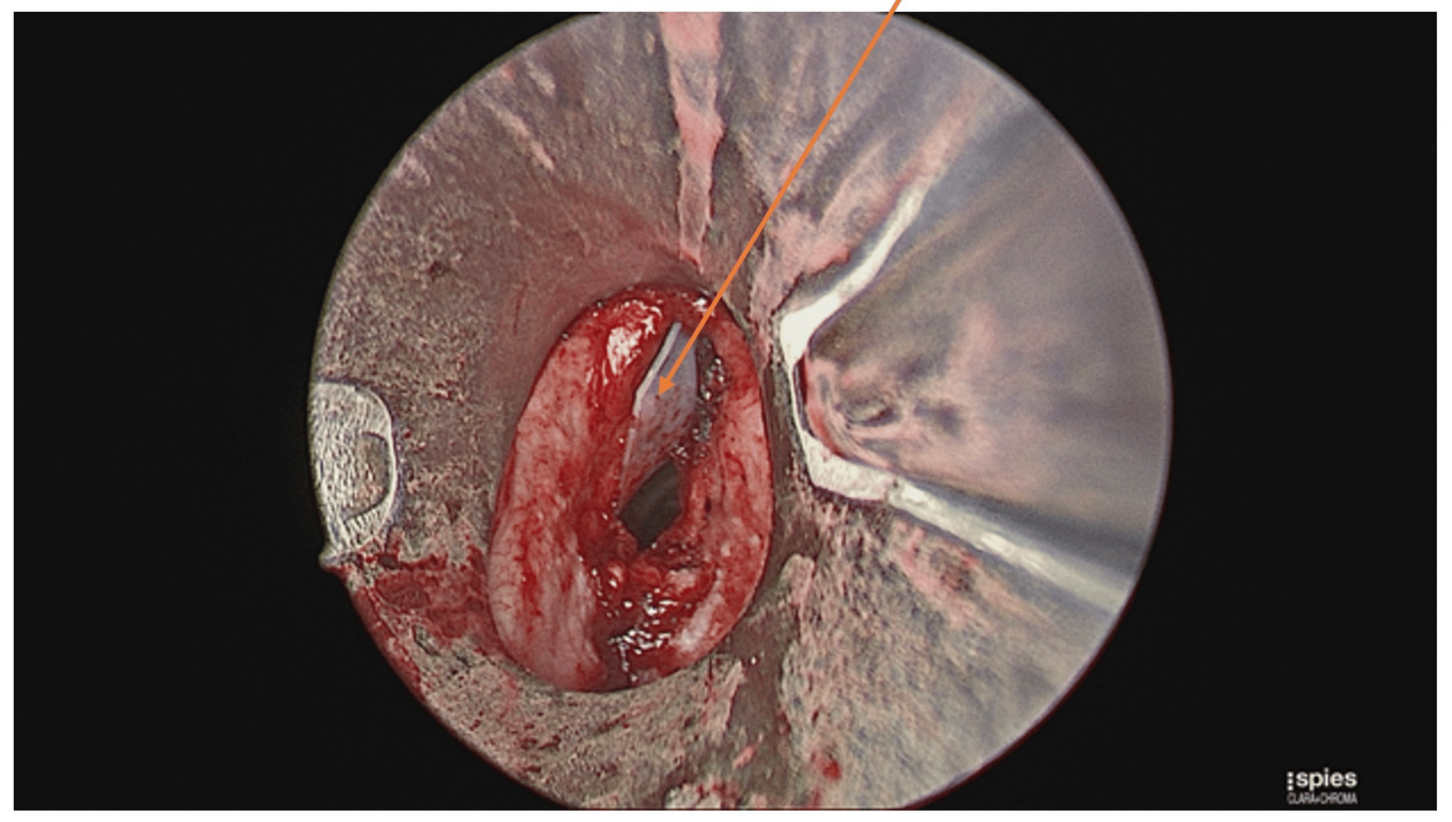
Insertion of the keel stent. The arrow is showing the keel stent.

The patient underwent an urgent microlaryngobronchoscopy with laser debulking of the laryngeal mass (Figures 2, 3) and insertion of a keel stent (Figure 4) under general anaesthesia. The purpose of the keel was to prevent the formation of any stenosis. He was discharged two days later, with a follow-up in two weeks’ time. Prior to this presentation, the patient has undergone six trans-oral laser resections of the larynx for recurrent presentations with laryngeal fibromatosis.

Biopsy samples were taken during microlaryngobronchoscopy and debulking. Three processes were evident in the histology slides, which included, firstly, fibromatosis changes, secondly, squamous proliferation, and, thirdly, spindle cell carcinoma. The histology was compared to those from previous biopsies and it was concluded that there was a malignant spindle cell transformation of his previous laryngeal fibromatosis. The histology findings were discussed in the ENT multidisciplinary team (MDT) meeting, and a total laryngectomy with central neck dissection and primary trachea-oesophageal puncture was recommended. Pathology results from the total laryngectomy confirmed laryngofibromatosis with squamous cell carcinoma. Following MDT discussion, no adjuvant therapy was recommended.

## Discussion

Aggressive fibromatosis, also known as desmoid tumour, is a rare (0.03% of all tumours), benign proliferative disease which commonly develops in muscle connective tissue, fasciae and aponeuroses [3]. Despite having similar characteristics as fibrosarcoma (a malignant tumour), desmoid tumours are considered benign due to their inability to metastasis [5]. They do, however, have a high rate of recurrence even after surgical treatment (25-65%) [4].

The aetiology of aggressive fibromatosis is unknown. They can occur sporadically, whilst, in some cases, they have been associated with familial neoplastic syndromes [2,11]. They are more common in females and amongst patients between 15 and 60 years of age [3]. The symptoms depend mainly on the size and location of the lesions and often occur as result of pressure on surrounding structures such as nerves and vessels [10].

Treatment of aggressive fibromatosis depends on the size and location of the tumour as well as the patient’s age and general health. Surgery is the mainstay of treatment [3,11]. In cases where surgery is not a suitable option, radiotherapy or chemotherapy can be considered [11]. So far, most reported cases of head and neck desmoid tumour have been managed by hemi- or total laryngectomy (Table 1) [4].

**Table 1:** Management of the previous eight cases of laryngeal fibromatosis Adapted from Shinohara et al. [4] M, male; F, female; R, right; L, left; LMS, laryngo-microsurgery; CO2, carbon dioxide; NA, not applicable

Case	Year of report	Age	Gender	Origin	Initial treatment	Recurrence	Final treatment
1	1989	67	M	Epiglottis	Excised by laser CO2 under LMS	Yes	Total laryngectomy
2	1994	65	M	Anterior commissure	Hemi-laryngectomy	Yes (6 months)	Total laryngectomy
3	1999	25	F	L Ventricle	Excisional biopsy under LMS	Yes (8 weeks)	Spontaneous regression
4	2001	75	M	Bilateral glottis-infraglottis	Total laryngectomy	No (5 years)	NA
5	2011	47	M	R false vocal cord	Hemi-laryngectomy	No (5 years)	NA
6	2016	67	M	R vocal cord	LMS	Yes (1 year)	Near-total laryngectomy
7	2018	65	M	R vocal cord	Laser cordectomy and ventriculotomy	Yes (15 years)	Hemi-colectomy with post-operative radiotherapy
8	2018	72	M	R vocal cord	Total laryngectomy	No	N/A

We described an atypical case of aggressive fibromatosis in a male, which manifested as laryngeal fibromatosis. Our patient presented with hoarseness of voice and dyspnoea - symptoms described in the previous eight reported cases [4-6]. Differential diagnoses for laryngeal masses more commonly include papilloma, granulomatous infections and myxoma [5]. Diagnosis of desmoid tumours, as in this case, is made histologically. They pathologically consist of identical and well-differentiated fibroblasts and fibrocytes. The histological findings in this case described the laryngeal lesion as hyperplastic mucosa with hyperkeratosis and collagenous tissue [4-5].

Considering the patient’s general health and the fact that the lesion was confined to the vocal cords, initially, trans-oral laser resection was deemed the best option for the patient. The high recurrence rate of aggressive fibromatosis despite surgical interventions and therefore the need for further surgical intervention was discussed with the patient. The patient underwent trans-oral laser resection performed six times since the time of his first diagnosis and had a symptom free period of eight years.

In this case, the biopsy showed that the laryngeal fibromatosis had developed into a spindle cell carcinoma. So far, no such case has been reported. Spindle cell carcinoma is a rare, biphasic tumour [12]. Histologically, it is comprised of malignant squamous and spindle cells [13]. Diagnosis of spindle cell carcinoma can be difficult if the squamous cell component cannot be histologically identified [13]. Common sites for this carcinoma include the larynx, oral cavity, pharynx and tonsils. Sarcomas are especially rare in the head and neck region; thus, spindle cell carcinoma should always be considered as a differential for lesions in this region [10]. It is more common in males aged 60 to 70 years. The aetiology of spindle cell carcinoma has been strongly associated with smoking and alcohol consumption and less commonly with radiation [12]. In this case, the patient was diagnosed at an atypical age (in his early 80s). He was, however, an ex-smoker and drank 40 units of alcohol a week, but with no history of radiotherapy. Distal metastasis is uncommon; however, regional lymph node metastasis has been reported in 25% of cases [13]. The metastatic foci often contain either only malignant squamous cells or both malignant squamous and spindle cells [13]. They very rarely have only malignant spindle cells. In our case, a staging CT scan did not show any metastasis.

A study conducted by Thompson et al., looking at 187 cases of spindle cell carcinomas of the larynx, reported that patients who were surgically managed had better prognosis compared to those who had radiotherapy alone [9]. In our case, the patient’s history and biopsy findings were discussed at the ENT MDT. The patient’s cancer was staged at T3 N0 M0, and, therefore, total laryngectomy was advised.

Spindle cell carcinoma is reported as an aggressive tumour with a high recurrence rate. Its prognosis depends largely on the location and appearance of the tumour, radiotherapy history and whether it has metastasised [9,12].

## Conclusions

We present a rare case of laryngeal fibromatosis that progressed to spindle cell carcinoma. This case underscores the critical importance of regular follow-up in such patients not only due to the high recurrence rate associated with aggressive fibromatosis but also because of the potential, albeit unusual, malignant transformation. Given the rarity of both aggressive fibromatosis and spindle cell carcinoma, evidence regarding the optimal management of these conditions remains limited.
